# Exploring Positive Deviant Behaviors Among High-Risk Pregnant Women With Different Birth Outcomes: A Qualitative Study From Palwal, India

**DOI:** 10.7759/cureus.91584

**Published:** 2025-09-04

**Authors:** Sonia Maurya, Krati Dixit, Shahil Attri, Reema Mukherjee, Vinod K Anand, Sarmila Mazumder, Barsha Gadapani Pathak

**Affiliations:** 1 Implementation Science, Society for Applied Studies, New Delhi, IND; 2 Community Medicine, Indian Council of Medical Research (ICMR), New Delhi, IND; 3 Department of Global Public Health and Primary Care, Centre for Intervention Science in Maternal and Child Health, Centre for International Health, University of Bergen, Bergen, NOR

**Keywords:** adequate antenatal care, early neonatal death, haryana, health-seeking behavior, high-risk pregnancy, india, positive deviance approach, stillbirth

## Abstract

Introduction

India has set the goals to reduce stillbirths (SBs) and neonatal deaths by 2030. However, despite continued efforts to improve maternal health, these adverse outcomes remain a concern in many regions. This highlights the importance of identifying strategies that are both community-acceptable and sustainable. This study explores positive deviant (PD) practices among pregnant women who, despite high-risk pregnancies (HRPs), achieved healthy birth outcomes - unlike many of their peers.

Methods

This qualitative exploratory study was conducted to explore PD practices among HRP women in Palwal district, Haryana, India. It focused on identifying what enabled some of these women to achieve positive birth outcomes, in contrast to their peers who experienced SB or early neonatal death (END). A total of 28 in-depth interviews (IDIs) were conducted with women with HRPs, categorized as women with live births (LBs) (N = 14), SBs (N = 9), and ENDs (N = 5). Data were analyzed thematically using Colaizzi’s seven-step method, and emerging themes were further interpreted using the "Theory of Planned Behavior" (TPB) framework to explore the role of individual attitudes, subjective norms, and perceived behavioral control in shaping maternal health behaviors.

Results

The PD women reported proactive health-seeking behavior, balanced nutrition, culturally rooted physical activity, and safe sleep positions, often supported by Accredited Social Health Activists (ASHAs), husbands, and family members. In contrast, women with SB or END outcomes faced delays, adverse traditional practices, partial information, and barriers in healthcare seeking. Family dynamics - especially the influence of mothers-in-law - strongly shaped decision-making around diet, supplements, and place of delivery. Higher perceived behavioral control enabled some women to navigate constraints and seek timely care.

Discussion

The findings highlight broader health and well-being challenges faced by pregnant women in low-resource settings. Physical vulnerabilities were often heightened by emotional stress, limited autonomy, and lack of support. Despite these challenges, some women showed agency by prioritizing their health and seeking timely care. These PD women adopted practices such as balanced nutrition, early antenatal care (ANC), and culturally accepted behaviors that contributed to healthy outcomes. Their actions offer valuable lessons that can be promoted through community awareness campaigns and health education materials. This study suggests the need for an integrated approach beyond physical care, including mental health support, respectful communication, and active family and community engagement.

Conclusion

To reduce preventable SBs in low-resource settings, maternal health interventions must go beyond clinical care to foster family-inclusive, culturally sensitive, and psychosocially supportive environments. Strengthening health worker capacities, aligning traditional practices with evidence-based care, and empowering women with accurate information, emotional support, and greater control over health choices are essential to promote positive health-seeking behavior.

## Introduction

Globally, the stillbirth rate (SBR) and neonatal death rate (NDR) were estimated at 13.9 and 17 per 1,000 live births (LBs), respectively, with India alone accounting for over one-third of these cases [1,2]. Stillbirths (SBs) and early neonatal deaths (ENDs) remain a significant yet under-prioritized public health concern, with profound physical, emotional, and financial consequences for families [3]. Despite progress in reducing under-five and neonatal mortality, SB prevention has received comparatively less attention - particularly in low- and middle-income countries (LMICs), where access to quality antenatal and delivery care remains limited [4].

India’s national policy framework, through the India Newborn Action Plan (INAP), aligned with the WHO Every Newborn Action Plan, commits to achieving single-digit SBR and NMR (<10 per 1,000 births) by 2030, with all states expected to meet this target by 2035 [5]. However, progress toward these goals is hindered by inconsistencies in case definitions, classification systems, and reporting mechanisms, resulting in wide variability in national estimates. For instance, the Sample Registration System (2020) reports an SBR of 3 per 1,000 births, while other national-level surveys report rates as high as 12.4 per 1,000 births [6,7]. This discrepancy reflects a fragmented data ecosystem, which undermines evidence-based planning and intervention.

The existing literature on SB and neonatal mortality has predominantly focused on biomedical determinants such as maternal obesity, hypertensive disorders, infections, and intrauterine growth restriction [8,9]. While these clinical risk factors are critical, they fail to fully account for the broader social, behavioral, and structural determinants that shape maternal and neonatal health outcomes, especially within resource-constrained settings. An emerging body of evidence suggests that maternal health-seeking behaviors, decision-making autonomy, intra-household power dynamics, and social support systems significantly influence pregnancy and perinatal outcomes [10,11].

To understand this better, the present study adopts the positive deviance (PD) approach, a behavior- and community-based change model that identifies individuals or groups who, despite facing similar risks and resource constraints, demonstrate better-than-expected outcomes [12]. The underlying premise of the PD approach is that within any community, there exist individuals who engage in uncommon but successful behaviors or strategies that enable them to overcome shared challenges [13]. PD has been effectively applied in various domains, including child nutrition, infectious disease control, and education, to identify sustainable, locally acceptable solutions to complex social problems [12-14].

In this context, the study aims to explore the application of the PD approach among high-risk pregnant (HRP) women in Palwal district, Haryana, a region with a persistently high SBR of 12 per 1,000 births [15,16]. Specifically, it seeks to identify the behaviors, coping mechanisms, and social practices that enabled certain HRP women to achieve positive pregnancy outcomes - defined as LBs without adverse neonatal events - despite facing comparable clinical and contextual risks as their counterparts who experienced SB or END. By systematically investigating these "positive deviants," this study aims to identify contextually grounded, community-informed, and potentially scalable practices that can inform the design of future interventions aimed at reducing perinatal mortality in similar settings.

## Materials and methods

Study design and setting

The present qualitative exploratory study was conducted in the Palwal district of Haryana, India, as a sub-study of an ongoing implementation research (IR) project titled Strategies to Help in Optimal Pregnancy Outcomes and Reduce Stillbirths in India (SHRiSTI) project. This SHRiSTI project aims to develop an optimized model of a comprehensive intervention package and delivery strategies to reduce SBs, and is also part of a larger IR initiative in low- to moderately-performing districts in India [16]. The Ethics Review Committee of the Centre for Health Research and Development, Society for Applied Studies, granted approval for this study (approval no. SAS/SOP/ERC/008T4). This sub-study was conducted to identify the PD practices that already exist among pregnant women and promote healthy pregnancy and birth outcomes. The PD approach was employed with the aim of identifying protective behaviors among HRP women with LB outcomes, despite facing similar complications as their peers with SB and END. The study also included descriptive quantitative data on socio-demographic factors to contextualize participants’ backgrounds and their access to healthcare services in Palwal.

The Palwal district has a population of over 1 million (77% rural), with significant gender (sex ratio: 879/1,000) and educational disparities, especially among women, with a literacy rate of 54.23%. The key maternal health challenges include an SB rate of 12/1,000 births [16], low antenatal registration (46.2% in the first trimester), and a high prevalence of low birth weight (LBW) (25.8%) [1]. The district health infrastructure comprises Primary Health Centres (PHCs) (N = 19), Community Health Centres (CHCs) (N = 5), Health and Wellness Centres (HWCs) (N = 89), and approximately 1,100 Community-Level Health Workers (CLHWs), such as Accredited Social Health Activists (ASHAs), Auxiliary Nurse Midwives (ANMs), and Anganwadi Workers (AWWs) [15].

Operational definitions

Stillbirth (SB)

SB refers to the birth of a fetus with no signs of life at or after 28 weeks of gestation. This includes the absence of breathing, heartbeat, or movement at birth.

Early Neonatal Death (END)

In this study, END refers specifically to the death of a live-born infant within the first 24 hours of life. This subset of ENDs is critical due to its association with intrapartum complications and immediate postnatal care quality.

High-Risk Pregnancy (HRP)

An HRP is defined as any pregnancy where the mother or fetus has an increased risk of adverse outcomes due to pre-existing medical conditions (e.g., hypertension, diabetes), obstetric complications (e.g., anemia, previous SB), or sociodemographic risk factors (e.g., teenage pregnancy, short birth intervals, low socioeconomic status). This categorization is based on the Government of India’s antenatal risk stratification guidelines under the Reproductive, Maternal, Newborn, Child, and Adolescent Health (RMNCH+A) framework [17].

Positive Deviants (PDs)

In this study, PDs were the women with an HRP who achieved an LB outcome despite facing similar clinical and contextual risks as their peers. Their unique but replicable behaviors and practices are the focus of PD inquiry.

Study participants

A purposive sampling strategy was used to recruit HRP women who delivered between March and May 2025. The study blocks were identified based on performance metrics of institutional delivery and quality of antenatal care (ANC), divided into three categories: a good-performing block (Hodal), moderately performing blocks (Dudhola and Alawalpur), and a low-performing block (Hathin) of the study district. Eligible participants were identified from PHCs and CHCs within these blocks, and a line list of all women who delivered (N = 863) was prepared, including their HRP status and birth outcomes. Later, the identified HRP women were followed via telephonic interviews by field supervisors with a graduation degree to confirm their HRP status and birth outcomes, i.e., LB, SB, and END. Out of the total confirmed HRP cases (N = 87), a separate list was prepared for different birth outcomes, i.e., LB (N = 58), SB (N = 19), and END (N = 10). The screening of SB and END cases was done using the WHO’s Verbal Autopsy Tool by trained field supervisors with a B.Sc. Nursing degree. Further, women who resided in Palwal for at least six months pre-conception were eligible, while women with incomplete contact information, who returned to their maternal homes post-delivery, or who declined participation were not included in this study. The final in-depth interviews (IDIs) were conducted with HRP women with different outcomes until data saturation was achieved. The total number of IDIs conducted has been categorized below under three groups: Group 1: HRP with LBs (N = 14); Group 2: HRP with SBs (N = 9); and Group 3: HRP with ENDs (≤24 hours) (N = 5).

Data collection

Semi-structured IDIs were conducted using a pre-structured IDI guide, following the national guidelines and reports [18,19], along with findings from the formative research. The IDI guide explored ANC visits, nutrition, danger sign awareness, delivery preparedness, and postpartum practices. It aimed to capture a wide range of domains relevant to HRPs, including the antenatal phase, intrapartum phase, and postnatal phase.

Antenatal Phase

Registration timing, ANC visit frequency, essential ultrasonography (USG) scan, micronutrient supplementation (iron and folic acid (IFA)/calcium), tetanus immunization, nutrition and dietary practices, rest and workload, danger sign awareness, weight monitoring, sleep positions, physical activity, and management of high-risk conditions (e.g., anemia, hypertension, gestational diabetes mellitus).

Intrapartum Phase

Facility preference, birth preparedness - including transport and financial arrangements - skilled birth attendants, birth complications, and decision-making dynamics were all important factors considered in this phase.

Postnatal Phase

The phase explored postpartum care practices, early and exclusive breastfeeding, newborn care, and maternal recovery.

The contextual relevance and clarity of the IDI guide were ensured through repetitive, iterative refinement via field pre-testing and expert review. Content validity was assessed by testing content clarity, logical sequencing, cultural appropriateness, and the coverage of relevant findings. This process included pilot interviews conducted with a small group of women from a non-study area who shared similar socio-demographic and obstetric characteristics with the target population. This approach was chosen to ensure that the women selected for testing the questions were not the same as those included in the final study, while still ensuring they had similar backgrounds and experiences to the actual participants.

The pilot testing was conducted by the lead social scientist, who was also responsible for conducting the main interviews. This continuous involvement ensured consistency between tool development and data generation. Feedback from pilot participants, along with structured debriefing meetings involving qualitative research experts, was used to revise the phrasing, sequencing, and framing of questions to enhance clarity, flow, and depth of responses. The final IDI guide, refined through these multi-step processes, is provided in Table 5 (see Appendix I).

All interviews were conducted at the participants’ homes to provide a comfortable and familiar setting. To maintain privacy and confidentiality, family members were respectfully requested to allow the participant to speak alone during the interview. This approach was informed by insights obtained from the pilot study, which showed that the presence of others could inhibit honest responses and sharing. In cases where privacy was difficult to secure, the interview team made flexible arrangements, such as requesting a quieter space or rescheduling, to minimize disturbances and safeguard the participant's comfort and confidentiality.

Given the sensitive nature of pregnancy-related experiences, special attention was paid to the emotional well-being of respondents during the interviews. A response guide was prepared for the interviewers, with recommended responses for distress management during the interviews. This response guide for distress management was developed collaboratively by the research team, drawing upon established psychosocial support frameworks and context-specific insights from formative research. It was piloted with a small subset of participants to assess its appropriateness and effectiveness, and, based on feedback from both pilot interviews and field investigators, it was iteratively refined for clarity, cultural relevance, and practicality in field conditions. Following this refinement, the finalized responses were used throughout the study to provide participants with emotional support in case they experienced distress or discomfort. If participants showed signs of emotional distress during the interviews, interviewers paused the discussion and provided appropriate support, following the procedures outlined in the response guide for distress management, which is provided in Table 6 (see Appendix II). This ensured that participants felt safe and supported throughout the interview process. After each interview, participants were thanked for their time, and follow-up care or counseling was offered, if and when necessary.

The interviews were conducted by a trained female social scientist with a PhD in Medical Anthropology, with a specialization in women’s health, who had extensive experience in qualitative research, particularly in maternal and child health. She was supported by a female project coordinator, holding a Master’s in Public Health (MPH), and trained in qualitative data collection techniques. While the social scientist facilitated the interviews, the project coordinator took detailed notes and documented non-verbal cues - such as hesitation, distress, or expressions of confidence - to add interpretive depth during analysis. Each interview lasted approximately 40 to 60 minutes, depending on participant responsiveness and comfort.

Throughout the data collection phase, the research team conducted regular debriefing sessions every two days. These sessions were held between the field teams and a medical doctor with public health expertise. The purpose of these sessions was to review field notes, identify emerging themes, assess consistency in participant narratives, and refine the interview guide if needed.

Data analysis

All interviews were audio-recorded, transcribed verbatim, and translated into English by the same researchers who conducted the interviews, ensuring consistency and contextual fidelity in the translation process. Data analysis followed a two-stage thematic analysis approach that combined both inductive (data-driven) and deductive (theory-driven) techniques [20].

**Figure 1:**
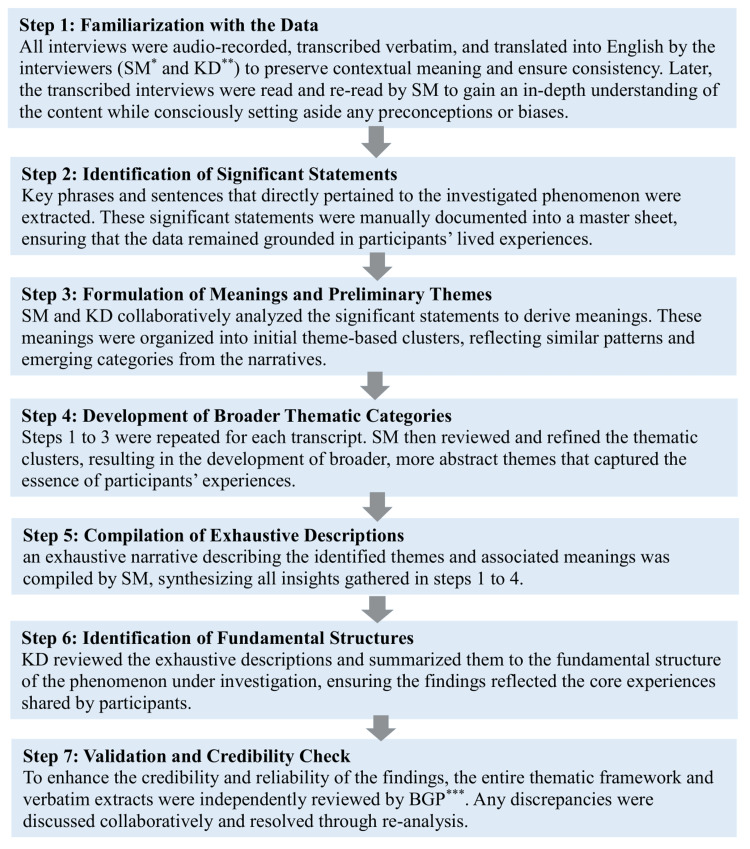
Modified Colaizzi’s Seven-Step Method for Data Analysis SM: Sonia Maurya*; KD: Krati Dixit**; BGP: Barsha Gadapani Pathak*** The initial coding was done manually due to regional language constraints. Final thematic organization was conducted using NVivo software (version 15.1.1), ensuring systematic documentation and traceability.

In the first stage, an inductive analysis was conducted, where themes were derived from the raw narratives without any pre-imposed theoretical framework. This allowed for the emergence of rich, grounded insights based on the lived experiences of HRP women. The thematic analysis was conducted by following Colaizzi's seven-step method to understand participants’ experiences by identifying key statements, finding their meanings, grouping them into themes, and cross-checking with IDIs to ensure the findings were accurate [21]. This method consists of a structured pathway of seven steps, which aids in the identification of core themes from raw data. Initially, in the first step, the transcripts were read multiple times by Sonia Maurya (SM) to become deeply familiar with the content, setting aside any personal biases or assumptions. In the second step, significant statements and relevant narratives were manually identified and documented in a master sheet. During the third step, SM and Krati Dixit (KD) collaboratively analyzed these statements, organizing them into preliminary theme-based clusters. In the fourth step, SM refined and reviewed the clusters to develop broader concepts and thematic categories. Steps 5 and 6 involved generating comprehensive descriptions, carried out by SM and KD, respectively. The final and most critical step involved an independent review by Barsha Gadapani (BGP), who reviewed and cross-checked the themes and verbatim. All the steps of Colaizzi's seven-step method are provided in Figure 1.

Any discrepancies in interpretation were resolved through discussion and reanalysis. Although initial processing was conducted manually due to the regional language used in the transcripts, all coding and thematic organization were subsequently carried out using NVivo software (version 15.1.1; QSR International, Melbourne, Australia) to ensure systematic documentation and consistency.

**Figure 2:**
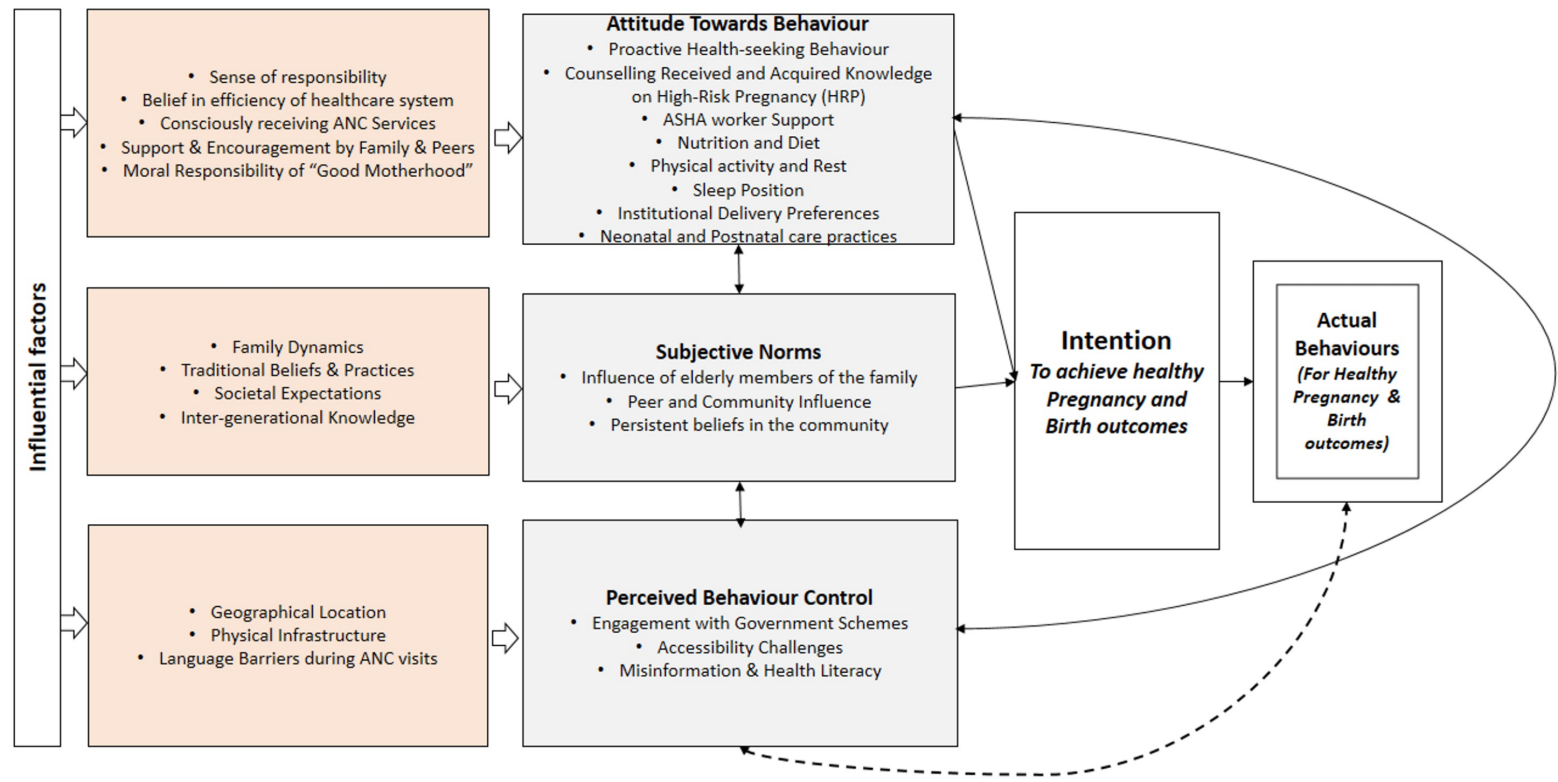
Modified Theory of Planned Behavior Framework Based of the Positive Deviance Practices Emerged in the Qualitative Thematic Analysis ANC: Antenatal Care; ASHAs: Accredited Social Health Activists

In the second stage, a deductive lens was applied using the Theory of Planned Behavior (TPB) to interpret and structure the findings within this broader psychosocial framework [22]. TPB was selected because it is widely used in public health research to explain how individual attitudes, perceived social pressures, and perceived control beliefs influence health behaviors. This approach helped in understanding relevant practices followed by pregnant women with HRP. A diagram of the modified TPB framework is provided in Figure 2.

The final themes were categorized under the three core domains of TPB: (I) behavioral beliefs, which refer to personal attitudes toward pregnancy-related behaviors and their expected outcomes (e.g., beliefs about food, rest, or ANC care); (II) normative beliefs, which involve perceived social norms and expectations from key influencers such as family members, community elders, and health workers; and (III) control beliefs, which encompass perceived internal and external facilitators or barriers to performing health-promoting behaviors (e.g., mobility restrictions, financial constraints, or CHW support).

## Results

Descriptive participant characteristics

Among the 28 participants, the mean age was 25.78 years (±6.19), with 20 (71.42%) aged 18-25. The mean age at first conception was 20.17 years (±2.28), with two (7.14%) women conceiving before the age of 18. One-third (9, 32.14%) had received no education, while 19 (67.85%) were literate. The majority of participants, i.e., 20 (71.42%), were living in joint families. Regarding reproductive history, nine (32.14%) were multigravida, and five (17.85%) had C-sections, with 23 (82.14%) delivering vaginally. More than half of the women (19, 67.85%) gave birth in private healthcare facilities (Table 1).

**Table 1:** Socio-Demographic and Reproductive Health Characteristics of the Study Population

Characteristics	N = 28	Mean (±SD)
	Count (%)	
Age (years)		
18-25	20 (71.42)	25.78 (6.19)
26 & above	8 (28.57)	
Age at first conception		
≥18	26 (92.85)	20.17 (2.28)
<18	2 (7.14)	
Family structure		
Joint	20 (71.42)	-
Nuclear	8 (28.57)	
Education		
Literate	19 (67.85)	-
Illiterate	9 (32.14)	
Mode of delivery		
Vaginal	23 (82.14)	-
C-section	5 (17.85)	
Delivery facility		
Public	9 (32.14)	-
Private	19 (67.85)	
Gravida		
Primiparous	10 (35.71)	-
2nd & 3rd	9 (32.14)	
Multigravida	9 (32.14)	

Qualitative findings

The IDIs were analyzed using the TPB framework under the behavioral beliefs (attitude), normative beliefs (subjective norms), and control beliefs (perceived control). Narratives emerged from IDIs were compared across three groups: HRP women with LB, SB, and END.

Attitude Towards Behaviors (Behavioral Beliefs)

Proactive health-seeking behavior: Women in the LB group described consciously seeking early ANC consistently throughout their pregnancies. Some of them shared that they made visits twice a month, underwent blood and urine tests, completed both routine (at least three timely ultrasounds) and Level II ultrasounds, and took iron, calcium, and folic acid tablets as prescribed. The research team observed that these women reflected a clear awareness of the importance of timely and continuous health monitoring, particularly among those with secondary education. 

In contrast, women in the SB and END groups reported delayed or irregular ANC, sometimes missing visits entirely due to feelings of helplessness, financial constraints, lack of family support, or low perceived urgency.

Counseling received and acquired knowledge on HRP: Several women with LBs mentioned they had received counseling about their HRP status during ANC visits. They were informed about complications such as cord loops, abnormal fetal presentation, low amniotic fluid, and a history of bad obstetric history (BOH). As a result, many chose to deliver in higher-level facilities or private hospitals.

Women in the SB and END groups often reported receiving inadequate or no counseling. Some shared that, even though they regularly visited facilities and underwent scans, they were not informed about complications. A few stated that they learned about issues like a cord around the neck only after labor had started, leading to multiple referrals and delays.

Nutrition and diet: Women in the LB group emphasized the importance of good nutrition during pregnancy. Many shared that they incorporated foods like milk, fruits (particularly pomegranate and apples), soaked dry fruits, and green leafy vegetables into their diets. Their dietary choices were influenced by advice from healthcare providers and family elders.

In contrast, women with SB and END food practices had more varied diets. While some did follow similar regimens, others reported avoiding certain items, such as “papaya” and “rice” based on cultural beliefs. Restrictions on foods considered “hot” or potentially harmful - such as papaya or certain spices - were common. In some cases, these women had poor intake of iron-rich foods or fluids. A few women consumed inappropriate items, such as “Nakkash,” which is paper inscribed with spiritual spells and consumed throughout pregnancy in the belief that it will ensure a healthy pregnancy.

Physical activity and rest: Women in all groups described remaining physically active during pregnancy, but the intent behind these actions varied. Women in the LB group often framed physical activity as beneficial for facilitating labor and fetal movement. They also described balancing work and rest, often resting after completing household chores.

In contrast, some women in the SB and END groups described engaging in strenuous activities, like agricultural work and carrying firewood, without adequate rest and hydration, while others restricted their movement out of fear or exhaustion. Only a few women in these groups explicitly linked physical activity to better outcomes.

Sleep position: Some women with LBs reported adopting side-sleeping positions, especially on the left side, based on advice or personal comfort, which they felt improved sleep and reduced abdominal pain. Few women shared that they provided an elevated platform of soft clothes below their abdomen while sleeping sideways to reduce belly discomfort and to comfort their developing fetus. All women, regardless of birth outcome, avoided sleeping straight due to discomfort.

However, women in the SB and END groups more often slept on the right side, and did not commonly mention adjusting sleep positions for comfort or pregnancy improvement.

Institutional delivery preferences: Women in the LB group often made advance decisions to deliver in private facilities or district hospitals, prioritizing hygiene, doctor availability, and timely care. These choices were influenced by personal experiences, family preferences - especially mothers-in-law - and medical advice. Factors such as better quality care, 24-hour doctor access, essential tests, and newborn care units motivated their selection of private facilities, often with strong family support for this decision.

In contrast, women in the SB and END groups frequently experienced multiple referrals during labor, and some first visited the traditional birth attendant for delivery. Many began labor at home or sought care at primary facilities with inadequate resources, leading to delays. These women often lacked awareness about selecting suitable facilities for HRPs and did not have peer support or advice on institutional delivery.

Neonatal and postnatal care practices: Women in the LB group followed regular postnatal care, including exclusive breastfeeding, timely immunization, and follow-ups with ASHA workers, adhering to medical advice.

In contrast, women in the END group often deviated from recommendations, using traditional pre-lacteal feeds like "Janam Ghutti". In this group, the incidence of newborn infections - particularly pneumonia - was higher compared to the LB group. Women in the SB group rarely discussed postnatal care, as the loss of their child and associated grief affected their engagement in such routines. The emerging themes and verbatim under the behavioral beliefs of TPB are provided in Table 2.

**Table 2:** Emerging Themes and Verbatim Under the Behavioral Beliefs of TPB Group 1: Women with high-risk pregnancies and live birth outcomes; Group 2: Women with high-risk pregnancies and stillbirth outcomes; Group 3: Women with high-risk pregnancies and early neonatal death outcomes. TPB: Theory of Planned Behavior; PCOS: Polycystic Ovary Syndrome; ASHA: Accredited Social Health Activists; CHC: Community Health Centre

TPB Domain	Sub-theme	Group 1	Group 2	Group 3
Attitude Towards Behaviors (Behavioral Beliefs)	Proactive Health-Seeking Behavior	“I visited the doctor in the second month as soon as I got to know about my pregnancy, and got my first USG done in the third, and then in the sixth month.”	“I had low Hb but did not get any injections or tests. I thought that it will be cured itself.”	“I did not visit any doctor or take any medicine after I got to know about my pregnancy in the month of May…I just did not visited there is no particular reason behind it… I was completely fine…So I didn’t feel like visiting hospital”
		“I went to the public facility for my check-ups in the fourth, fifth, and seventh months. The doctor gave me my second vaccine dose and advised a Level II ultrasound in the fifth month. Which was not available in the government hospital, so with the help of my brother, I had this ultrasound scan in the 5th month. I kept track of my appointments and went on my own in the village’s government hospital whenever needed. I wanted to be sure that everything was okay with my current pregnancy. Even if my mother-in-law didn’t understand why so many visits were necessary… I knew from what I had learned from my sister-in-law and doctor has also advised me to be careful this time to avoid the death of the baby again, which happened in my last pregnancy.”	“I couldn’t go for check-ups regularly... sometimes I just felt too tired or sad. And with the household work, it was hard to manage time.”	“I got only one injection during my pregnancy that too in the 8th month…because I visited the hospital in the 8th month due to fever, then the doctor gave me one injection. I don’t know which injection it was…”
		“I visited twice a month and never missed my medicines.”	“We didn’t have enough money. The travel and tests cost too much, so I went only when really needed, like in emergency only.”	“I didn’t feel any problems during pregnancy, so I missed some visits... I thought everything was fine.”
		“I went to the public facility for my check-ups in the fourth, fifth and seventh month, I got my second dose of vaccine and level II USG done in the fifth month.”	“My mother-in-law said, I have given birth to my all children without any this hospital visit or medicine…She said it is not required to take any medicine or visit hospital because it, unnecessary medicine case harm only… So no one supported me to go.”	“I wanted to go, but with small children at home and no one to help, it was difficult.”
		“I visited the doctor in the second month as soon as I got to know about my pregnancy and got my first USG done in the third and then in the sixth month.”	-	“After my husband lost his job, we didn’t have money. So I delayed going to the doctor.”
		“I started going as soon as I knew I was pregnant. I would go every two weeks to the clinic. They checked my blood, urine, and did the sonography. The doctor said it’s important, so I didn’t miss.”	-	-
		“I completed all the scans on time. Doctors told me three ultrasounds are needed and one detailed one, I did all of them. I also took all the medicines, iron, calcium, folic acid, every day.”	-	-
		“Since I studied till 10th standard, I knew a little about pregnancy care. I made sure to follow what the nurse told me, for the health of my baby.”	-	-
	Counseling Received and Acquired Knowledge on High-Risk Pregnancy	“There was a hospital near my mother’s home, so I shifted there in the early months of my pregnancy and I stayed there till delivery. From there, I visited the hospital regularly because previously I had a C-section, so the doctor advised me to visit frequently.”	“No one told me about the cord. I showed the report but was not informed. My baby was okay in the first hospital we visited… the heartbeat was low but baby was alive but in the second hospital when we entered they did not check anything because in was night and doctors were not available, then in the third hospital which was a private hospital they checked the heartbeat of the baby and declared my baby dead.”	“Even though I went for checkups, they never said anything was wrong. Only during delivery they said the baby was not coming down properly.”
		“I was told the cord was around the neck, so we went to a private hospital directly.”	“I went for scans, but no one told me there was any problem. Later, during labor, they said the baby had a cord around the neck…If they have informed me I might have visited some other better private hospital.”	“They (doctors) didn’t tell me about any complications. If I had known, maybe we could have gone earlier to a bigger hospital.”
		“I was undergoing my PCOS treatment and I followed exactly what my doctor recommended and did not miss my supplements and vaccines.”	“I didn’t know there was a risk. They didn’t explain anything during my visits.”	“I only found out after the baby was born that there was some problem with breathing, no one had warned us.”
		“During my checkups, the doctor told me the baby had less water (amniotic fluid), so they advised me to deliver in the city hospital.”	“We found out too late that there was low water and the baby was weak, by the time they referred us, it was already serious.”	-
		“I was told about the risks because of my previous miscarriage. So this time, I was very careful and went to a better hospital.”	-	-
	Nutrition and Diet	“Every day, I had soaked almonds and fruits, as my ASHA advised.”	“They told me not to eat papaya or pineapple, so I avoided those.”	“I was told to avoid papaya and too much milk, but I didn't like the smell and taste of the milk and most of the food items, so I didn’t eat much...I prefer having the pickle and chapatti during my pregnancy… almost all the days after the 3rd month.”
		“The doctor advised me to avoid lifting heavy objects and not to consume food items which have a warming effect in the initial month.”	“I didn’t feel like eating much. Also, we couldn’t afford fruits every day.”	“I mostly ate what was made at home. Sometimes I avoided certain things, like brinjal, because elders said it’s not good in pregnancy.”
		“I start my day by having a glass of water and then take a glass of milk, later I eat some fruits and then for lunch I consume vegetable, dal, rice, chapati, salad and curd and then later in evening I used to drink coconut water followed by chapati and vegetable in dinner. Along with that I used to take buttermilk and curd with sugar during the day and also some soaked sprouts and nuts.”	“I heard that some foods are ‘hot’ and can harm the baby, so I avoided spices and some vegetables.”	“I didn’t drink much milk; it made me feel heavy.”
		-	“When my pregnancy was confirmed, I also took medicine from the 'Maulvi.' I visited him in my 3rd, 5th, 6th, and 8th months. He gave me 'Nakkash' (paper pieces with spiritual writings) to dissolve in water and drink for protection…I was having 'Nakkash' daily… I didn’t feel the need to have any other fruits and milk… my in-laws can't afford the expense of fruits.”	-
		“I took chapati, vegetable and buttermilk and also ate fruits like pomegranate and apple.”	-	“No one told me about what to eat or not eat.”
		“I used to start my day by having a glass of water and then taking a glass of milk, later I ate some fruits and then for lunch I consumed vegetable, dal, rice, chapatti, salad and curd. Later in evening, I used to drink coconut water followed by chapati and vegetable in dinner. Along with that, I used to take buttermilk and curd with sugar during the day, and also some soaked sprouts and nuts.”	-	-
	Physical Activity and Rest	“Walking helped me feel the baby move more.”	“I worked till late because of marriage at home, even when I was unwell. No one advised me to take a rest.”	“I kept doing all the heavy work at home because my husband was away.”
		“I used to walk for 30 minutes daily on the terrace.”	“I had to work in the fields till late in pregnancy, it depends on the season, if the field have crops and are ready, then we have to cut those crops, other it might get ruined due to rain… we didn’t have a choice.”	“I didn’t know resting was important, I worked as usual till delivery.”
		“I didn’t do any household work for three months post my delivery.”	“I used to carry water and firewood... sometimes I was too tired, but there was no one to help.”	“I stopped moving much because my body would ache a lot, I just stayed inside.”
		“I used to sleep for 3 to 4 hours a day after sending my children to school and finishing my work.”	“I was in my ninth month, but I still had to go to the fields. There was no one else to do the work. I cut grass, carried bundles of firewood on my head, and walked long distances. Sometimes, I wouldn't even drink enough water because I’d be out all day. I was tired all the time, but I didn’t think it would affect the baby, I just thought, this is how it is for women like us. A few days later, I started having pain. We reached the hospital late. They said the baby had no heartbeat. I still wonder, maybe if I had rested a little more, maybe if someone had helped me… things would be different."	-
		“I could feel the baby’s movement when I used to walk.”	-	-
		“My mother-in-law advised me to walk and to do floor cleaning while sitting by, ensuring me that it would help me deliver my baby normally. So I made sure to stay active during the day, sweeping, cooking, and doing all the household work. I heard from neighborhood women as well, it helps the baby move well and makes delivery easier. But did everything but carefully and also took a rest once I finished everything in the daytime. It's about keeping a balance, not overdoing it…. Because doing anything over might harm.”	-	-
	Sleep Position	“My mother-in-law said to avoid sleeping straight; I felt better on the side.”	“I usually slept on my right side, no one told me about any position.”	“I am habitual of sleeping mostly on the right side. I didn’t know it made any difference.”
		“I started sleeping on my left side in the last months… Nobody told or advised me… I did it myself because I felt comfortable in that position, and also kept the soft cloth elevation below my belly side by thinking it would be better for the baby, and honestly, I felt more comfortable that way. When I slept straight, my back and stomach would hurt, so I just turned to the side. It helped me sleep better, too.”	“I didn’t think much about sleep positions. I just slept however I could.”	“Throughout my pregnancy, I usually slept on my right side. It just felt natural to me; I’ve always slept that way. No one, not the doctor or nurse, ever said anything about which side is better during pregnancy. I didn’t know it made a difference.”
		“I felt comfortable sleeping sideways because if I sleep straight I feel a pressure in my abdomen while getting up.”	“It was uncomfortable to sleep straight, so I turned to the side, mostly the right.”	“Nobody told me about which side to sleep on, I just found whatever was comfortable.”
		-	-	“I avoided sleeping on my back because it felt heavy, but didn’t pay attention to left or right.”
	Institutional Delivery Preference	“My in-laws insisted on a private hospital; we planned to visit private hospital in advance.”	“We went to a CHC, but there were no doctors, and I was referred twice.”	“During my pregnancy when I was not able to feel the baby’s movement one day I asked my mother-in-law to call the ‘local dai’ who stays in our village.”
		“Right from the start, my mother-in-law and Husband said we should go to the private hospital this time. She had seen what happened during my first delivery in the public center; there was no doctor at night, and the place wasn’t clean. This time she said, ‘We won’t take any risks with your or the baby’s life.’ I also wanted a place with good doctors who were available if something went wrong. We all agreed. She accompanied me and walked with me for the checkups.”	“My baby did not cry after the delivery because he got stuck down (birth canal) during delivery, and I think it is very wrong as the nurse asked my family members to go out and then said that she cannot take the guarantee for the baby’s survival.”	“The dai suggested me to come to some clinic where she was working when her night shift will start at 7 pm.”
		“These days, deliveries are risky, and anything can happen. In private hospitals, the doctor sees you quickly, the baby gets care if needed, and the place is better managed. Money comes and goes, but life doesn’t come back. That’s why we all said, no compromise this time.” ~Mother-in-law	“They were pushing the baby downwards with pressure during the delivery.”	“My labor started late at night. We called the local 'dai' because she had helped with births in our village for years. I trusted her, and so did my mother-in-law. She tried for hours, but the pain became too much, and the baby wasn’t coming. Only in the morning did they decide to take me to the public hospital in the village, but there was no doctor there. From there, we were sent to another hospital. It was too late. The baby was born, but didn’t cry.”
		“My elder daughter was born by C-section so at the time of this baby we didn’t think about money and we went to the private hospital because in the nearby public facility they don’t do delivery by C-section.”	“That evening, I felt something was wrong. The baby hadn’t moved like usual. I told my mother-in-law, and she said we should wait, that sometimes babies rest more in the last days. The next morning, when I still didn’t feel anything, she called the 'dai' (Traditional birth Attendant) from our village. The 'dai' checked and said things seemed fine, but I didn’t feel right. We finally went to the health center, but they said they couldn’t help and sent us to the district hospital. By the time we reached, they said the baby had no heartbeat. I keep thinking, what if we had gone earlier?"	-
	Neonatal and Postnatal Care	“I gave only breast milk for six months. We followed the vaccine card carefully.”	“There was no baby... I didn’t do anything after delivery.”	“We gave 'Janam Ghutti.' It’s our custom, though the nurse said not to.”
		“After delivery, I made sure to give only breast milk for the first six months, nothing else, not even water. The nurse and ASHA didi explained why it’s important, and I followed it. We kept the vaccine card safe and took the baby for every injection on time. If I had any doubt, I would ask the ASHA. She would come and check on us, and that gave me confidence.”	“I couldn’t think about anything after the loss.”	“I did not give only breast milk, we used honey and ghutti too.”
		“The anganwadi worker told me to keep a hand under the baby’s head while feeding.”	“We didn’t talk with any doctor or ASHA worker about after delivery, I just stayed at home.”	“I didn’t take the baby for any injections; the baby was very weak.”
		“I gave only breast milk from the beginning. The ASHA came and told me how to do it.”	“After the delivery, we didn’t speak to any doctor or ASHA worker. There was nothing to ask… the baby was gone. I just stayed at home, didn’t step out much. No one mentioned check-ups or anything else. I was sad and crying continuously, and honestly, no one around me said it was needed.”	“We gave 'Janam Ghutti' right after birth, it happens in our community’s tradition. My mother-in-law said we’ve always done it. The nurse did mention that we should avoid it, but we didn’t listen much. The baby had loose motions after that, but we didn’t think it was serious at the time.”
		“I took my baby for all the injections as the nurse said, didn’t miss any.”	-	-
		“ASHA came home and checked the baby. I followed all her advice.”	-	-

Subjective Norms (Normative Beliefs)

Family influence: Support from family members - especially mothers-in-law and husbands - emerged as a major theme across groups. Women with LBs described how their family members reminded them to attend ANC visits, helped them access supplements, or encouraged them to rest.

In the SB and END groups, family influence was sometimes restrictive. Women shared that they were discouraged from taking iron tablets due to fears about large babies, or were asked to avoid certain foods or check-ups unless problems were visible.

ASHA worker support: Women in the LB group often highlighted the role of ASHA workers in providing timely advice, reminders for medicines, arranging transport, or interpreting scan reports. They described ASHA workers as accessible and responsive.

Women in the SB and END groups also mentioned interactions with ASHAs but described receiving more general advice. Some expressed confusion over schemes like "Janani Suraksha Yojana (JSY)" or "Pradhan Mantri Matru Vandana Yojana (PMMVY)," or services, despite contact with frontline workers.

Peer and community influence: Women with LBs often reported that their peers and community members followed formal healthcare practices, like regular ANC, immunizations, and institutional deliveries. Observing this motivated them to adopt similar behaviors, reinforcing the importance of medical care for safe births.

In contrast, women in the SB and END groups lacked strong peer influence toward formal healthcare. Their peers often relied on traditional practices, home remedies, or spiritual coping, and held fatalistic beliefs about pregnancy outcomes. This reduced their likelihood of following medical advice or seeking timely care. Traditional practices, such as using "Lohban," "Guggal" fumigation, and consuming "Nakkhas," often delay formal medical intervention. The emerging themes and verbatim under the normative beliefs of TPB are provided in Table 3.

**Table 3:** Emerging Themes and Verbatim Under the Normative Beliefs of TPB Group 1: Women with high-risk pregnancies and live birth outcomes; Group 2: Women with high-risk pregnancies and stillbirth outcomes; Group 3: Women with high-risk pregnancies and early neonatal death outcomes. TPB: Theory of Planned Behavior; JSY: Janani Suraksha Yojana; ASHA: Accredited Social Health Activists

TPB Domain	Sub-theme	Group 1	Group 2	Group 2
Subjective Norms (Normative Beliefs)	Family Influence	“My mother-in-law was the one who guided me the most. She would remind me to go for check-ups and tell me what to eat, like more green vegetables and fruits. She had seen many pregnancies in her time, so I trusted her. She used to say, ‘don’t forget medicine, these medicine will provide strength to you and the baby.’ Whatever I needed, my husband helped me and provided me whatever I asked…sometimes even doing the household work so I could rest.”	“My mother-in-law said not to take too many iron tablets, the baby will become too big and cause problems, like C-section.”	“My husband said checkups are unnecessary unless something is seriously wrong.”
		“I used to get all the information from my mother-in-law only about birth preparedness and diet during pregnancy.”	“Whenever I said I wanted to go for a check-up, my husband would say, ‘Why waste money if everything seems fine?’ He thought doctors were only needed when there was a big problem. So we didn’t go regularly. Even when I felt a little uneasy, we waited at home.”	“In the beginning, I took the iron tablets they gave me. But after a few weeks, my mother and Jethani (husband’s elder brother’s wife) said that too much medicine is not good and makes the baby too big, which might cause an operation (Cesarean section). So I stopped. No one else insisted, so I just stopped taking any medicine."
		"My mother-in-law told me to take my tablets regularly and eat well.”	“They told me not to go for checkups unless I was feeling sick.”	“My family said taking too many medicines is not good, so I stopped after a while.”
		“My husband took me to the health center for checkups.”	“In our house, they said certain foods are not good, so I avoided them.”	“No one told me to go for checkups, they said, if ‘you’re fine, no need.’”
		“The family reminded me to rest after finishing housework.”	-	“My mother-in-law believed eating ghee and sweets was better than taking tablets.”
	ASHA Worker Support	“ASHA came regularly and explained everything in our language.”	“She told me something about a form, but I don’t know which scheme.”	“ASHA told me to take medicines, but I was confused about when to go to the hospital.”
		“When I found out I was pregnant, we called the ASHA didi the same day. She picked up right away and said, ‘Don’t worry, I’m here to help you.’ After that, she called before every vaccination, reminded me to collect my iron and calcium tablets, and even advised us to go to the doctor because she could not interpret the ultrasound reports. Once, when my husband couldn’t get leave from work, she arranged transport for my check-up and accompanied me. I have her contact number, I would just call her for any information.”	“ASHA came sometimes, but only said to go for checkups, nothing more.”	“We heard about some government money (JSY), but didn’t know how to get it.”
		“We informed the ASHA about the pregnancy in the beginning over the call and then she used to call me at the time of the vaccination.”	“We didn’t understand much about the schemes, no one explained clearly.”	“ASHA came to the house, but didn’t say much about anything.”
		-	“The ASHA came once or twice and spoke about some scheme, something about filling a form… but I didn’t understand what it was for. No one explained it clearly, and I didn’t ask again. We also heard that the government gives some money after delivery, but no one told us how to apply or if we were even eligible.”	-
	Peer and Community Influence	“In our colony, most women go for hospital check-ups and deliveries. I saw my neighbor going for her third-month scan and coming back with pictures of the baby, they were all talking about it. Another didi in our lane told me how the nurses gave her iron tablets and injections that helped her during pregnancy. My sister-in-law also said, ‘Now no one gives birth at home, go to the hospital, it's better.’ So I followed what they were doing, and I think that helped me and my baby.”	“People said complications are fate. They didn’t encourage checkups.”	“I took 'Nakkhas' because others in my family did it; they said it’s good for the baby…it will save the baby from evil eyes and spirits.”
		In our village, most women go for checkups and take the tablets, so I also did the same.”	“In our community, many women use home remedies first, then visiting hospital… Visiting hospital is the last option.”	“No one in my area talks about vaccines or checkups.”
		“My friends told me they delivered in the hospital. I also wanted to go there.”	“When I started feeling uneasy in the seventh month, my neighbors and relatives said it’s just part of the process, it will pass. No one talked about going to the doctor. Instead, they told me to burn 'Lohban' in the evening and keep 'Nakkhas' brought by mother-in-law from a 'Maulvi' under my pillow to protect the baby from 'buri nazar' (evil eye). Even when I wanted to go for a check-up, they said, ‘What’s the use? Whatever is written will happen.’ So I followed what they said.”	“I don’t trust hospital people, they just waste our time and do nothing I have visited during my last pregnancy, I just waited and got no treatment and medicine…We prefer traditional treatments and medicine from 'Maulvi sahib' (traditional healer).”
		“Everyone in our home, my sister-in-law and aunties have given vaccines to their babies its common now. Hospital staff themselves provide the vaccination to the baby which is required to be given within 24 hours and then they explain us to take information about the aaganwadi workers of our areas”	“My neighbors said to use 'Guggal' smoke to remove evil, I followed that in the evening and very next day in morning I lost my baby.”	“I took 'Nakkhas' because others in my family did it; they said it’s good for the baby…it will save the baby from evil eyes and spirits. My mother-in-law gave me 'Guggal' to burn in the room every evening to keep away evil spirits.”
		-	“People in my family and neighborhood believe if something bad has to happen, it will happen, medicines can’t change that.”	-

Perceived Behavioral Control (Control Beliefs)

Engagement with government schemes: Women with LBs reported applying for and receiving financial assistance through government schemes, often with ASHA support, and expressed satisfaction with the help received during pregnancy and delivery.

In contrast, women in the SB and END groups were aware of the schemes but did not apply due to a lack of clear information, uncertainty about procedures, and confusion over the application process, which left them unsure about how to access the benefits.

Accessibility challenges: Transportation and distance to health facilities were challenges for all groups. Women in the LB group, despite limited transport or absent husbands due to work, often walked long distances or were accompanied by female relatives or neighbors to reach care.

In contrast, women in the SB and END groups faced greater barriers from distance, poor roads, transport costs, and lack of support. Many stayed home during advanced pregnancy and relied on traditional remedies or spiritual coping due to these obstacles.

Misinformation and health literacy: Participants across groups encountered misinformation. Women in the LB group actively sought clarification from ASHAs or doctors, and based their decisions on verified advice.

In contrast, women in the SB and END groups often acted on fears or rumors - such as concerns about tetanus shots - and relied on inaccurate information from peers or family without seeking clarification. The emerging themes and verbatim under the perceived control beliefs of TPB are provided in Table 4.

**Table 4:** Emerging Themes and Verbatim Under the Perceived Controlled Beliefs of TPB Group 1: Women with high-risk pregnancies and live birth outcomes; Group 2: Women with high-risk pregnancies and stillbirth outcomes; Group 3: Women with high-risk pregnancies and early neonatal death outcomes. TPB: Theory of Planned Behavior; ASHA: Accredited Social Health Activists

TPB Domain	Sub-theme	Group 1	Group 2	Group 2
Perceived Behavioral Control (Control Beliefs)	Engagement with Government Schemes	“I submitted the form and followed up until I got the money.”	“We heard about the scheme, but no one told us how to apply.”	“I knew there was a scheme, but I didn’t understand how to get it.”
		“The ASHA worker told me about the form for the government scheme. I filled it out and gave it to her. When it didn’t come the first time, I followed up again. I submitted the form multiple times and followed up until I got the money.”	“I didn’t understand the process, so we didn’t apply.”	“We were not clear about how to get the money; no one explained it properly.”
		-	“I thought only if the baby is alive, they give money, so we didn’t ask.”	“ASHA came after delivery, but I was confused about what documents were needed.”
		-	-	“I heard there is a scheme, but I don’t know how to do the paperwork.”
	Accessibility Challenges	“I walked to the hospital with my mother when my husband was busy.”	“It’s too far, and buses don’t go there. So I stayed home and prayed to god for my wellbeing.”	“No one was available to take me to the hospital, so we tried home remedies first.”
		“Even though my husband was away, my sister-in-law came with me to the health center.”	“The road was very bad, we couldn’t go in time.”	“The clinic was too far, I couldn’t manage to go in the last month.”
		“We walked to the center, it was far, but I didn’t want to miss the checkup.”	“We didn’t have money for transport, so I stayed home.”	“Transport cost was too high, we waited too long before going.”
		“There was no transport, so I managed by going with neighbors.”	“When the labor pains started, it was too late to arrange a vehicle.”	“Once, I said I needed to go for a check-up in 5th or 6th month, but no one at home was available to take me. I couldn’t go alone. I asked the ASHA worker, but she said she couldn’t arrange a vehicle. So, I didn’t go. In our village, especially for daughters-in-law, going out alone is not considered appropriate. Back in my maternal home, I used to accompany my sister-in-law for hospital visits, but here, the rules are different, and I am unfamiliar with the area. If someone from the family is available to go with me, then I step out. There is no strict restriction on going out, but I must have someone accompany me.”
		“When I started getting pain, my husband was away for work. I didn’t want to wait, so I told my mother. We walked slowly to the hospital, it took us almost 40 minutes. She kept saying, ‘Just a little more, we’re close now.’ I was tired, but I knew I had to reach. We didn’t have a vehicle, but still, we managed. The doctor said it was good that we came on time.”	“When the pain started at night, we called for a vehicle, but it didn’t come for a long time. My brother-in-law borrowed a bike, and we somehow reached the nearby center. But there they said, ‘We can’t handle this, go to the district hospital.’ We had to go again in another vehicle. By the time we reached, I was feeling dizzy and weak. They checked and said the baby had no heartbeat. I don’t know… maybe if we had reached earlier, something could have been done.”	-
	Misinformation and Health Literacy	“ASHA told me clearly, so I ignored what neighbors said.”	“I didn’t take the tetanus injection; they said it causes problems.”	“People said the calcium tablets cause swelling, so I stopped taking them.”
		“The doctor used to give me at least 20 minutes and told me to include nutritious food like seasonal vegetables and fruits, and the ASHA also advised me to walk every day and always called me to remind me about the vaccination. Even now, she does this for my baby’s immunization.”	“Some women said the injection is risky and could harm my pregnancy… such as I could have lost my baby.. so I avoided it.”	“I was scared of the hospital, they said women have operations unnecessarily.”
		“At first, I was scared of the tetanus injection, but the ASHA Didi explained and I took it.”	“Family said not to take iron, it will make the baby too big.”	“I didn’t ask anyone about what is right or wrong, I just followed what others in the house said.”
		“Some people said not to take too many tablets. I asked the doctor, and he said it is safe.”	“I heard that too many medicines and injections can harm the baby and can also cause miscarriage, so I didn’t go much.”	-
		“I heard many things, but I only followed what the nurse or ASHA told me.”	-	-

## Discussion

The findings of the present study assessed the PD behaviors under three major themes: individual attitudes, subjective beliefs, and perceived control behavior of TPB. It was found that women’s attitudes were shaped not only by individual beliefs but also by subjective norms and control beliefs during the antenatal, intrapartum, and postpartum periods. The strong intention to achieve a healthy child motivated many women to adopt behaviors that aligned with medical recommendations, such as regular ANC, improved nutrition, physical activity, appropriate sleep position, institutional delivery, and maternal and neonatal care during the postnatal period. However, these individual attitudes were continuously shaped by prevailing cultural practices, intergenerational knowledge, and the influence of family members - particularly mothers-in-law and husbands - as well as peers in the neighborhood.

The majority of women demonstrated a proactive approach toward ANC, aligning with studies from similar low-resource settings where maternal health engagement was framed as a moral and familial duty [23]. However, this behavior was stratified by birth outcomes; those who experienced LB showed higher compliance with recommended supplements (iron, calcium, and folic acid), whereas non-compliance was more common among women with SB or END. This is supported by previous research [24], which indicates that adherence to IFA is positively associated with a healthy birth.

The timely counseling and early decision-making regarding an appropriate birthing facility played a crucial role in managing complications that may arise during pregnancy and intrapartum as well. Women informed about their HRP status and advised to seek care early at well-equipped facilities (neonatal intensive care units, ventilators, and skilled pediatricians) mostly had LB. On the contrary, delays in referral and repeated referrals, especially in the context of fetal distress, were commonly reported among cases of SB and END, consistent with previous findings [25]. This highlights the need for a timely and effective referral system that depends on several factors, including adequate resources, reliable transportation infrastructure, social support, and informed decision-making. Yet, gaps in risk identification, management, counseling, and institutional support persisted, and challenges were also noted in the literature on referral delays and limited health literacy [26]. Nutrition-related practices showed an interplay between medical advice and traditional beliefs. Many women integrated nutrient-rich foods (dry fruit laddoos, leafy vegetables, and milk) into their diets, consistent with medical guidance for reducing complications such as pre-eclampsia [27]. Simultaneously, cultural taboos - such as avoiding “hot” foods like papaya - persisted, often mediated by elder family members. Similarly, postpartum diets, including ghee- and jaggery-based laddoos and water-rich fruits, reflected deliberate self-care practices rooted in cultural knowledge, which require thoughtful integration with medical interventions. This coexistence of medical advice and culturally rooted food restrictions illustrates a duality in dietary behavior. Such patterns reflect findings from India and other South Asian contexts, where food practices during pregnancy are shaped by symbolic meanings and health beliefs [28].

Physical activity patterns during pregnancy further illustrated this triangulated influence. While many women associated light activity (walking and household chores) with positive outcomes, those engaged in strenuous labor, such as agricultural work or wood collection during late pregnancy, faced higher risks of SB and END. Importantly, these behaviors were not merely a function of individual choice but were influenced by family expectations, financial constraints, and limited awareness of associated risks. Existing literature suggests that it is important to understand that, while light to moderate physical activity during pregnancy is generally good for health, doing very hard or tiring work can be harmful - especially for women with high-risk conditions like placenta previa and weak cervix (cervical incompetency) [29].

Sleep positions were based on individual choices and influenced by intergenerational wisdom, such as advice from the mother-in-law. Such intergenerational knowledge transfer is consistent with the findings of, who emphasize the cultural transmission of maternal knowledge in rural contexts [30]. Previous studies have linked back- and right-sided sleeping with a higher risk of SB, reduced fetal growth, LBW, and preeclampsia [31,32].

Crucially, the study found that healthcare decision-making was rarely autonomous. Instead, it was deeply influenced by social factors and communities. The authority of mothers-in-law, supported by their moral and experiential status, often influenced dietary practices, supplement adherence, and interpretations of medical interventions - sometimes supporting harmful practices, e.g., avoiding iron supplements to prevent perceived risks of macrosomia - consistent with previous findings [33,34]. Similarly, the role of husbands was prominent in financial decisions, transport arrangements, and the choice of delivery facility, yet was frequently mediated by maternal elders. This reflects a “triadic model of influence” that is particularly salient in rural and semi-urban India, where pregnant women must navigate the competing expectations of multiple family members [35,36].

Community norms also influence women’s health behaviors. Negative perceptions of public healthcare (overcrowded clinics and disrespectful staff) discouraged some women from engaging with ANC, despite geographical proximity to healthcare services. Moreover, beliefs prevalent in peer groups - such as fears about vaccinations or perceived benefits of private hospitals - strongly influenced choices of health facility and service utilization. In line with social network theory [37], these findings highlight the relational nature of behavioral control, where collective narratives either enable or constrain individual agency.

ASHAs played a pivotal role in mediating between formal health services and traditional belief systems. ASHA workers were often trusted for translating medical advice into local dialects or languages, providing culturally harmonious information. Women with LBs frequently reported proactive ASHA involvement and information received from her for early pregnancy registration, regular ANC attendance, and birth preparedness. However, variability in ASHA knowledge and communication quality was also evident, particularly among less-educated women who expressed a need for clearer, more actionable guidance - emphasizing the importance of enhanced training and structured health literacy interventions [38,39].

Women’s perceived behavioral control was shaped not only by personal motivation but also by structural enablers and constraints. Financial barriers, limited transport, and poor road conditions frequently restricted timely access to care. However, PD (women with LBs), despite similar constraints, demonstrated higher inherent motivation, resourcefulness, and support from peers or family members. These behaviors included proactive planning for the prior arrangement of local transport and prioritizing institutional delivery. These results highlight that healthy behaviors are not solely determined by external barriers but by the dynamic interaction of individual autonomy, social support, and health system preparedness.

Importantly, the preference for institutional delivery was influenced by a combination of factors, such as perceived safety and access to skilled care, encouragement by family members and ASHAs, and the ability to plan and afford healthcare. Women with greater education, financial stability, or stronger agency were more likely to make informed facility choices and prepare proactively for safe delivery [25]. However, some institutional deliveries still resulted in negative outcomes, often due to late referral or facility unpreparedness. Viewed through the lens of the Three Delays framework, these findings illustrate how delays in deciding to seek care (influenced by individual and family-level factors), delays in reaching appropriate facilities (such as repeated referrals), and delays in receiving quality care (night-time coverage gaps or limited readiness of facilities) together shape maternal and newborn outcomes. This highlights the need to address both timely care-seeking and systemic preparedness to ensure safe delivery.

Finally, misinformation circulating in communities - such as the belief that tetanus injections cause infertility - showed how prevalent community beliefs could undermine adherence to ANC and vaccination, consistent with prior studies [40]. Some participants described the information provided by ASHA workers to access financial entitlements such as JSY or PMMVY. This aligns with existing studies, which suggest that timely information and community health worker facilitation are critical determinants of successful scheme utilization in rural India [41]. However, even when awareness existed, incomplete or fragmented knowledge hindered full participation. This reflects the phenomenon of “partial empowerment,” where women have the intention and basic awareness to engage with services but lack the procedural clarity, confidence, or consistent institutional support to realize their goals [42-44]. 

Limitations

While this study offers valuable insights into PD practices among HRP women, certain limitations must be acknowledged. The research was conducted in a single district, intentionally selected for its high burden of SBs, neonatal deaths, and other adverse pregnancy outcomes. However, this localized focus may limit the transferability of findings to other cultural or regional settings. As with most qualitative studies relying on retrospective accounts, there is a possibility of recall and reporting bias, especially among participants who experienced emotionally distressing outcomes. Efforts were made to create a supportive environment to encourage accurate and open sharing. Additionally, some degree of social desirability bias may have occurred, with PD women possibly emphasizing favorable behaviors. Despite these limitations, the study provides a meaningful understanding of community-rooted maternal health behaviors that can inform future interventions.

## Conclusions

This study offers an understanding of PD practices among women, such as proactive health-seeking behaviors, culturally mediated dietary and physical activity practices, sleep position, and the powerful influence of familial and societal expectations on decision-making. Despite structural barriers such as limited healthcare access, misinformation, and infrastructural challenges, many women demonstrated adaptive agency and resilience, often supported by community health workers like ASHAs. Importantly, the study highlights the need for culturally sensitive, family-inclusive interventions that not only inform but also empower women through practical, accessible, and contextually relevant health choices and communication. Strengthening the role of frontline health workers, improving the clarity and consistency of information, and addressing infrastructural barriers are essential for enhancing maternal and child health outcomes. Ultimately, the findings suggest that improving maternal health in low-resource settings requires an integrated approach - one that respects relevant traditional knowledge while incorporating medical care practices to ensure every woman has both the intention and the ability to achieve a safe and healthy pregnancy.

## Appendices

Appendix I

**Table 5:** Guide for In-Depth Interviews With Pregnant Women on Practices Followed by Them During Pregnancy: A Positive Deviance Inquiry This In-Depth Interview Guide has been developed by the authors. Credits: Sonia Maurya and Barsha Gadapani Pathak

Guide for In-Depth Interviews With Pregnant Women on Practices Followed by Them During Pregnancy: A Positive Deviance Inquiry	
Introduction	
Thank you for taking the time to speak to me today. My name is (interviewer name), and I am one of the SAS team members. Before we begin, can I please confirm that you have received a copy of the study information sheet and consent form? As a reminder, this project aims to develop a package of interventions that will help to prevent deaths, by promoting coverage and quality of the healthcare required during the antenatal period and labor. This form will have questions to understand what practices have been followed during pregnancy that have led to the live birth, what happened during pregnancy, the description of the illness or accident, any complications, the type of care sought and treatment received, and any challenges faced by you in availing the health care services. This will help us in strengthening the health systems to prevent such tragedies from happening in future. There are no right or wrong answers. Everything you say will be treated confidentially and will not be shared with anyone outside of our study team. This interview will take approximately one hour, depending on how much you have to say. Can I please check if you are free now to talk for this amount of time? I would also like to record our conversation - so that I can capture your responses accurately, and so that I can listen to you rather than take many notes. Can I confirm you are happy for me to start recording? Thank you.	
Information to collect (before starting):	
Unique ID/RCH ID	
Age (in years)	
Education (completed years)	
Number of Children (live/abortion/dead/stillborn)	
Age and sex of the children	
Gestational age sex of the most recent birth	
Place of birth	
Date of the interview	
Unique ID/RCH ID	
Age (in years)	
Education (completed years)	
Antenatal period	
Q. Can you tell me about your pregnancy? How did you get to know about your pregnancy? How was your experience overall? (Any specific feeling & emotional change. Perception about her pregnancy: Were there any self-doubts about carrying a recent pregnancy?) How did you feel when she shared this news with her husband & family? How do they react?	
Q. Please, tell me about your experience with antenatal care. Did you attend antenatal care? How many times did you go? If yes, where do you go for this (Public/Private, level of facility) and reason? (Timing of the visit, accompanying person, purpose, experience, Gestational month, purpose of visit) If antenatal care was not attended, what reasons? (accessibility & affordability, multigravida, terrain, poor experience, behavior of healthcare staff)	
Q. Did you have an ultrasound? When and where did you go for an ultrasound? What did you learn from ultrasound reports? If you did not have any ultrasound, why?	
Q. Were you aware of any healthcare workers in your area? (Type of Interactions, support received)	
Q. Did the health worker identify any potential problems or conditions during your pregnancy and talk to you about being at high risk? If yes, what information and advice did you receive in ANC? (Counselling sessions, explained signs of high-risk pregnancy: anemia, hypertension, eclampsia, pre-eclampsia, STI, hypothyroidism, diabetes, etc.)	
Q. Did you face any complications during your pregnancy? (Nature of complications: Early pregnancy: spotting, dizziness, vomiting, diarrhea, fainting & weakness, APH (fresh blood or old blood); BP, diabetes, constant pain, sign of chloasma; Hypertensive disorders: PIH, Pre-eclampsia, eclampsia, Gestational Diabetes, STI, Hypothyroidism, Hepatitis B, HIV, Malaria). How were they managed? Have you needed any specialized care during pregnancy? If yes, how were they addressed? (Timely identification of any complications, management of pre-term labor, medicine in case of PROM; communication with healthcare providers, Hospital, self-care, traditional medicine, etc.)	
Q. Have you faced any health issues in the past? (Any chronic conditions, previous disease history & any surgeries, Menstruation cycle related issues- length of MC; irregularity; Infection, UTI, Anemia, Diabetes, thyroid, Blood pressure, etc.)	
Q. How do you feel about the interaction with healthcare providers? (Active listening, elaborate explanation of the health condition, paying respect, adequate time, friendly environment, privacy, infrastructure)	
Q. Who makes the decisions about your healthcare? How involved are you in these decisions?	
Q. Did you face any financial difficulties in accessing healthcare? If yes, how do these financial issues impact your ability to get the care you need?	
Q. Are there any cultural or social beliefs in your family that affect your healthcare during pregnancy? What, Why & How it affects you? How is it important from their point of view?	
Q. What would you suggest be done during pregnancy to better support women for birth preparedness and complication readiness?	
Q. What kind of support did you receive during your pregnancy? (Social support; Emotional support; financial support; Support in household chore: Kind of support by husband & other family members (including maternal family); Care)	
Q. What nutritional intake have you followed during the current pregnancy? (fruit intake, tea intake, number of meals, additional supplements)	
Q. According to you have time to rest during the pregnancy? (In day & night time, both- no. of sleeping hours & sleep position, leisure time, etc.)	
Q. What was your daily routine throughout your pregnancy? (Leisure time, Physical activity pattern, Yoga & exercise, etc.)	
Q. Are there any lifestyle habits you want to share? (Smoking (tobacco, pan-masala, etc.) and drinking (hookah, any alcoholic drink status, non-alcoholic drinks such as coke, limca, and other soft drinks)	
Q. Are you involved in any kind of work that contributes to your family financially? If yes (kind of work, Duration during pregnancy, working hours, lunch timings & no. of meals during the working schedule)	
During Intrapartum	
Q. Now I would like to ask you to tell me about the events of the day of the delivery. What happened that day? Please tell me in detail from the time you started experiencing pain or any complications you faced on that day or before. If birth at the Facility: Why did you go to the facility where you went to for birth? Did your husband or anyone else in the family have an opinion about where you were to give birth? Who made the decision? Why was it decided that you would give birth at this facility? Did you have difficulties in reaching the facility, e.g., was transport easily available, or a cost problem? Distance? Terrain? Any other barriers? (Quality of care: Skilled birth attendant, clean birth practices, timely identification of any complications, management of pre-term labor, medicine in case of premature rupture of membrane; Behavior of healthcare provider: Felt disrespect or ignored communication with staff, any specific incidents)	
Q. When you reached the facility, what action was taken by the healthcare providers/nurse before delivery? (Checked: Women's vitals, Fetal presentation, FHR at what interval, when was the last FHR taken before delivery, use of Doppler ultrasound)	
Q. Did a family member accompany you and support you during labour and childbirth? If yes, who accompanied or supported you, and how did they support you? Was it useful/helpful having them with you? Please tell me more. If birth at home: Why did you prefer to have a birth at home? Was this your decision or someone in your family? Why didn’t you go to a facility for birth? Who assisted with the birth, and what family members were with you during the birth? Do you know whether the person was a trained health worker (skilled birth attendant)? What support did you receive from family members?	
Post delivery	
Q. How did you feel at that time when you heard you had a stillborn or that you were going to have a stillborn? Have you heard about such babies in her community? (Her perceptions about such birth, if not the norm; any apprehensions regarding reactions by family, family support; any blame on her; any worries about the next pregnancy, etc.)	

Appendix II

**Table 6:** Response Guide for the Interviewers for Addressing Emotional Distress During the In-Depth Interviews This response guide tool for interviewers for addressing distress has been developed by the authors. Credits: Sonia Maurya and Barsha Gadapani Pathak

Response Guide for the Interviewers for Addressing Emotional Distress During the In-Depth Interviews	
Statement of Purpose: This document is designed to guide researchers on how to respond/react during the data collection process on a sensitive topic & scenarios. Sensitive topics are defined as topics having the potential to cause physical or psychological distress to participants or the researcher, such as sadness, anger, anxiety, and fear (Westland et al., 2024). The primary goal should be to ensure the emotional safety and dignity of the participant while still allowing them the opportunity to share their story if they wish. The interviewer’s role is to provide a compassionate, non-judgmental space and to respond appropriately if the participant becomes distressed.	
Suggestions while taking an interview, either formal or informal: Always listen more than you speak. An interviewer must use non-verbal support (gentle nods, soft tone). Avoid phrases like “I understand” unless you have experienced similar loss; instead, say “Thank you for trusting me with your story”. It is important to debrief yourself after emotional interviews by seeking help from your peers, most preferably the senior researchers who are part of the study. Smith expressed, “to interview and then leave someone in emotional distress without adequate support or safeguards is morally wrong” (Whitney and Evered, 2022)	
Expected Questions & Scenarios during the Data Collection	Suggestions & Statement for the Interviewer
Initiate the Interview with empathy, patience, and flexibility.	Before we begin, I want you to know that some of the topics we will discuss can be emotional. If at any point you feel uncomfortable, need a break, or would prefer not to answer a question, that is completely okay. Please feel free to let me know. Your well-being is very important to us.
Women are concerned about whether they will experience a similar pregnancy loss again in the future	“Yes, we understand your concern, and this is a valid question. I feel your obstetrician is the best person to ask this question since they understand your case better and they will help you better prepare for your next pregnancy.” “Haan hum samjte hai aapke jo sawal/chinta hai who sahi hai, lekin is barre mein aap jin doctor ko dikhate hai (obstetrician) unse puchange toh behtar hai, kyunki woh aapka case behtar tarekae sae samjte hai aur aapko aane waali pregnancy mein aisi dikat na ho uskae liyae taiyaar karenge”
Family members ask, “Now the baby is no more, how can this interview help us? We had visited the hospital a number of times, and nobody took care of us.”	“We understand that you are upset by your experience at the facility, but what information you give us today will help the government in preventing such deaths in the future by making improvements in the services provided during pregnancy and at the time of delivery.” “Hum sajmhte hai aapko asptaal mein dikkate aayi thi,lekin aaj jo aap jaankari humein denge usse sarkaar ko yeh samjne mein madad melegi ki garbwasta aur prasav kae dauraan dekhbaal mein kin cheejo mein sudhaar ki zaroorat hai.”
First-time pregnant women are generally more hesitant to talk about the death of the baby, compared to women who have had babies in the past. Other family members responded in her place. How to encourage and convince the family members so that we can get the woman herself to talk about her experience	Instructions for FIs - First, listen to everything the family members have to say respectfully. Then request them to let us talk to the woman (if possible, alone) because certain emotions and experiences that the woman has gone through, no one can explain these things better than her, and we would prefer to hear directly from her. Possible follow-up questions: What kind of emotions or experiences do you want to know about? “Because we want to understand her feeling of loss and ensure her well-being,” FI kae liyae nirdesh – “Pehle family members ki saari baatien samanpurwak sunnae. Phir unsae nivedan karein ki woh humein mahila sae baat karnein ki anumati de (yadi mumkin ho, toh akele mein) kyunki yeh bhavnayein aur anubhav jinse mahila gujri hai usse behtar koi aur nahi samjha payega aur hum siddhe usse sunna pasand karenge.” Sambhavit follow-up sawal- aap aisi kin tarah ki bhavnayein ya anubhav kae barrein mein janna chahte hai? "kyunki bacche ko khone ki bhavna jo mahila nae mehsoos kari hai, hum uski sthiti ko behtar samjhna chahte hai aur uski poori salahmati sunashchit karna chahte hai”
Sometimes family members (husband, father-in-law, and mother-in-law) say, “We can give you any information you need, the baby has passed away, we don’t want you to ask her pressing questions”. How to handle this situation?	In this case, we have to read the room, and we can approach it two ways - If the family prefers, it would be better if the visit takes place in a few days, as the woman will be in a better condition to answer questions. -We can ask the family member to also be present during the interview for their satisfaction. Aisi stithi mein, uss samay k mohol anusaar jawaab de, Agar parivaar chahta hai ki yeh visit thodae dino mein ho, taaki mahila sawaalo kae jawab dene kae liyae behtar stithi mein hogi. -hum parivaar kae sadasyo ko bhi unki santushti kae liyae interview kae dauraan upastit honae kae liyae keh sakte hai”
In some cases, the ladies say that when they got admitted for delivery, at that time, FHR was checked, and the nurse told them the heartbeat is present. But the baby was born stillborn.	Even at the time of delivery, there can be complications that can result in the baby being born stillbirth. Kai cases jinmein pregnancy kae dauraan koi dikkat nahi bhi hoti hai, unmein bhi chances hai ki delivery time pae dikkate ban jati.
In some cases, women show post-pregnancy symptoms, and they turn to the FA for advice	Yes, we understand your concern, and this is a valid question. In case symptoms are observed post-delivery you should consult your obstetrician since they know your case better and can help manage symptoms or provide treatment if required. “Haan, hum aapki chinta ko samjte hai, aur aapkae sawaal sahi hai. Yadi prasav kae baad lakshan dikhte hai, toh aapko apnae prasutti rog visheshagya sae paramarsh karna chahiye kyunki veh aapki pregnancy ko behtar jaante hai aur lakshano ko rokne mein madad kar sakte hai aur agar aavshyak ho toh upchaar pradaan kar sakte hai”
During the Interview: Key Signs of Emotional Distress Crying Becoming very quiet or withdrawn Shaking, breathing heavily, or visible distress Expressing feelings of guilt, anger, or hopelessness	Recognize that crying or emotional moments are normal and do not necessarily mean the interview must stop.
If the Participant Becomes Emotional	Allow the participant to set the pace and offer breaks if needed. Pause: Stop asking questions. Acknowledge their emotion: Gently recognize and validate their feelings. Offer comfort: Allow silence; offer tissues if available. Assurance of confidentiality: before resuming the interview. Give control back: Ask if they would like to pause, continue, or stop. Respect their decision: Never pressure them to continue.
Always prioritize the participant's well-being over completing the interview, but in case the participants don’t want to or are not in a state of sharing anything, either the interview should be stopped immediately, or the interviewer should show some empathy towards the participants by using the script provided for the interviewer	Interviewer Script: "I know this is very difficult to talk about. Please take your time." "It's okay to feel emotional. Would you like to take a break, have a moment, or continue when you’re ready?" "Thank you for sharing such a deeply personal experience. We can pause here if you would like."
If the Participant Wants to Stop	Thank them for their courage and time. Gently ask if they would like to share anything. Close the interview respectfully.
Closing Statement	"Thank you again for speaking with me today. I truly appreciate you sharing your experiences. Please remember to take care of yourself.”

## References

[1] Purbey A, Nambiar A, Roy Choudhury D, Vennam T, Balani K, Agnihotri SB (2023). Stillbirth rates and its spatial patterns in India: an exploration of HMIS data. Lancet Reg Health Southeast Asia.

[2] (2025). Levels and trends in child mortality: report 2023. https://policycommons.net/artifacts/11755691/levels-and-trends-child-mortality-report-2023/12646949/.

[3] Aggarwal N, Lahariya C, Sharma B (2023). Stillbirths in India: current status, challenges, and the way forward. Indian J Pediatr.

[4] Aminu M, van den Broek N (2019). Stillbirth in low- and middle-income countries: addressing the 'silent epidemic'. Int Health.

[5] Ministry of Health and Family Welfare (2014). Ministry of Health and Family Welfare, Government of India. India Newborn Action Plan (INAP) [Internet]. New Delhi: National Health Mission, Ministry of Health and Family Welfare; 2014 [cited. India Newborn Action Plan (INAP).

[6] Ministry of Health and Family Welfare (2025). Health management information system (HMIS) report 2019-2020. Health Management Information System (HMIS) Report 2019‑2020.

[7] Dandona R, George S, Majumder M, Akbar M, Kumar GA (2023). Stillbirth undercount in the sample registration system and national family health survey, India. Bull World Health Organ.

[8] Rosenstein MG, Cheng YW, Snowden JM, Nicholson JM, Caughey AB (2012). Risk of stillbirth and infant death stratified by gestational age. Obstet Gynecol.

[9] Neogi SB, Negandhi P, Chopra S, Das AM, Zodpey S, Gupta RK, Gupta R (2016). Risk factors for stillbirth: findings from a population‑based case‑control study, Haryana, India. Paediatr Perinat Epidemiol.

[10] Bhutta ZA, Das JK, Bahl R (2014). Can available interventions end preventable deaths in mothers, newborn babies, and stillbirths, and at what cost?. Lancet.

[11] Upadhyay UD, Dworkin SL, Weitz TA, Foster DG (2014). Development and validation of a reproductive autonomy scale. Stud Fam Plann.

[12] Sabale R, Pathak BG, Manapurath RM (2021). Utilizing "positive deviance inquiry" to explore factors influencing child health: a qualitative study. J Educ Health Promot.

[13] Lapping K, Marsh DR, Rosenbaum J (2002). The positive deviance approach: challenges and opportunities for the future. Food Nutr Bull.

[14] Foster BA, Seeley K, Davis M, Boone-Heinonen J (2022). Positive deviance in health and medical research on individual level outcomes - a review of methodology. Ann Epidemiol.

[15] (2025). Health management information system (HMIS) report 2023. https://hmis.mohfw.gov.in/.

[16] Pathak GB, Mukherjee R, Kandpal V (2025). Implementation research to develop an optimized delivery model for effective implementation of evidence-based interventions to reduce stillbirth in India: a study protocol. PLoS One.

[17] Ministry of Health & Family Welfare (2013). A Strategic Approach to Reproductive, Maternal, Newborn, Child and Adolescent Health (RMNCH+A) in India.

[18] (2025). National Family Health Survey (NFHS-5), 2019-21. https://dhsprogram.com/pubs/pdf/FR375/FR375.pdf.

[19] (2025). My safe motherhood: booklet for expecting mothers. https://nhm.gov.in/images/pdf/programmes/maternal-health/guidelines/my_safe_motherhood_booklet_english.pdf.

[20] Finlayson CS, Fu MR, Squires A, Applebaum A, Van Cleave J, O'Cearbhaill R, DeRosa AP (2019). The experience of being aware of disease status in women with recurrent ovarian cancer: a phenomenological study. J Palliat Med.

[21] Morrow R, Rodriguez A, King N (2015). Colaizzi’s descriptive phenomenological method. Psychol.

[22] Ajzen I (1991). The theory of planned behavior. Organ Behav Hum Decis Process.

[23] Hulton LA, Matthews Z, Stones RW (2000). A Framework for the Evaluation of Quality of Care in Maternity Services. Southampton: University of Southampton.

[24] Tolera C, Tafesse T, Dessalegn R, Amenu D (2024). Utilization of iron-folic acid supplementation and related factors in pregnant women in Leka Dulecha District, East Wollega Zone, western Ethiopia: the case study. Health Sci Rep.

[25] Koblinsky M, Matthews Z, Hussein J (2006). Going to scale with professional skilled care. Lancet.

[26] Bohren MA, Hunter EC, Munthe‑Kaas HM, Souza JP, Vogel JP, Gülmezoglu AM (2014). Facilitators and barriers to facility-based delivery in low- and middle-income countries: a qualitative evidence synthesis. Reprod Health.

[27] Pretorius RA, Palmer DJ (2020). High-fiber diet during pregnancy characterized by more fruit and vegetable consumption. Nutrients.

[28] Olajide BR, van der Pligt P, McKay FH (2024). Cultural food practices and sources of nutrition information among pregnant and postpartum migrant women from low- and middle-income countries residing in high income countries: a systematic review. PLoS One.

[29] Guinhouya BC, Duclos M, Enea C, Storme L (2022). Beneficial effects of maternal physical activity during pregnancy on fetal, newborn, and child health: guidelines for interventions during the perinatal period from the French National College of Midwives. J Midwifery Womens Health.

[30] Sam DL, Berry JW (2010). Acculturation: when individuals and groups of different cultural backgrounds meet. Perspect Psychol Sci.

[31] Wilson DL, Fung AM, Pell G (2022). Polysomnographic analysis of maternal sleep position and its relationship to pregnancy complications and sleep-disordered breathing. Sleep.

[32] Meneo D, Baldi E, Cerolini S (2024). Promoting sleep health during pregnancy for enhancing women's health: a longitudinal randomized controlled trial combining biological, physiological and psychological measures, Maternal Outcome after THERapy for Sleep (MOTHERS). BMC Psychol.

[33] Lassi ZS, Middleton P, Bhutta ZA, Crowther C (2019). Health care seeking for maternal and newborn illnesses in low- and middle-income countries: a systematic review of observational and qualitative studies. F1000Res.

[34] Inhorn MC, Patrizio P (2015). Infertility around the globe: new thinking on gender, reproductive technologies and global movements in the 21st century. Hum Reprod Update.

[35] Davis A, McCrimmon T, Dasgupta A (2018). Individual, social, and structural factors affecting antiretroviral therapy adherence among HIV-positive people who inject drugs in Kazakhstan. Int J Drug Policy.

[36] Tayal D (2019). Gender inequality, reproductive rights and food insecurity in Sub-Saharan Africa - a panel data study. Int J Dev Issues.

[37] Celentano DD (2010). Social networks and health: models, methods, and applications: by Thomas W. Valente. Am J Epidemiol.

[38] Scott K, Beckham SW, Gross M, Pariyo G, Rao KD, Cometto G, Perry HB (2018). What do we know about community-based health worker programs? A systematic review of existing reviews on community health workers. Hum Resour Health.

[39] Kok G, Gottlieb NH, Peters GJ (2016). A taxonomy of behaviour change methods: an intervention mapping approach. Health Psychol Rev.

[40] Mitchell SL, Schulkin J, Power ML (2023). Vaccine hesitancy in pregnant women: a narrative review. Vaccine.

[41] Agarwal S, Curtis SL, Angeles G, Speizer IS, Singh K, Thomas JC (2019). The impact of India's accredited social health activist (ASHA) program on the utilization of maternity services: a nationally representative longitudinal modelling study. Hum Resour Health.

[42] Randive B, San Sebastian M, De Costa A, Lindholm L (2014). Inequalities in institutional delivery uptake and maternal mortality reduction in the context of cash incentive program, Janani Suraksha Yojana: results from nine states in India. Soc Sci Med.

[43] Khan SU, Goldsmith AH, Rajaguru G (2021). Women’s empowerment over recreation and travel expenditures in Pakistan: prevalence and determinants. Ann Tour Res Empir Insights.

[44] Rai R, Singh R, Azim A, Agarwal A, Mishra P, Singh PK (2020). Impact of critical illness on quality of life after intensive care unit discharge. Indian J Crit Care Med.

